# Laser Treatment for Melanin Gingival Pigmentations: A Comparison Study for 3 Laser Wavelengths 2780, 940, and 445 nm

**DOI:** 10.1155/2020/3896386

**Published:** 2020-03-09

**Authors:** Manaf Taher Agha, Pavel Polenik

**Affiliations:** ^1^Charles University in Prague, Department of Stomatology (Pilsen), Prague, Czech Republic; ^2^Adjunct Faculty Member, Ajman University, Faculty of Dentistry, Ajman, UAE

## Abstract

The normal appearance of the gingiva is pink to light red, and this appearance may change due to many factors and might be noticeable causing aesthetic concerns. In the Gulf area, the gingival melanin pigmentation is of the main type of concern, and patients expect the pigmentation to be removed for aesthetic reasons. Many techniques have been used to remove the melanin pigmentation such as using surgical blades, diamond or ceramic burs, chemicals, and lasers. This study is comparing the results of three lasers (Er, Cr, YSGG 2780 nm, Diode 940 nm, and 445 nm) in the removal of melanin gingival depigmentation. Clinical outcome parameters including bleeding, wound healing, pain, duration of procedure, color improvement, patient satisfaction, and relapse rate after 2 years were assessed. *Conclusion*. Within the limitation of this study, all three wavelengths were fast, effective in peeling the pigmentations and well tolerated by the patients. The esthetical results were excellent, and the patients were highly satisfied. *Suggestion*. To have a bigger number of samples in future papers, and histological studies might be included to explore the different impacts of each wavelength on the gingival melanin pigmentation specifically and on the gingival tissue generally.

## 1. Introduction

The normal appearance of the gingiva is pink to light red, with stippling “orange peel texture.” The normal color depends on melanogenesis and distribution of melanin pigment, keratinization depth of epithelization, and vascularity of the gingiva [1–4].

Pigmentations of oral soft tissues for patients is dominantly an aesthetic problem as lifestyle, media, social, and community influence can affect people's perception of beauty, which has a big impact on the person's self-confidence and could affect their social interaction and careers. An example is melanin pigmentation of the gingiva which in the Gulf region became a real concern for the new generation specially females, seeking for an aesthetic solution to remove the pigmentation. Surprisingly, it has been noticed that in some parts of Africa, they do not like the pink gingiva and they have used dark tattoos to change the color; this explains the different beauty perceptions among societies in different parts of the world.

Pigmentations could be a sign of pathology; hence, it is always important to investigate the causes and reach to a clear diagnosis.

According to contemporary classification, oral pigmentation is a discoloration of the gingival/oral mucosa; and it may result from exogenous and endogenous factors [1–3].

Etiological factors are varied which include drugs, heavy metals, genetics, endocrine disturbances, syndromes as Albright's syndrome, Peutz Jegher's syndrome, and also in inflammation. Adverse habits such as smoking can also stimulate melanin pigmentation, and the intensity of pigmentation is related to the duration of smoking and number of cigarettes consumed [1, 2].

The pigmentation is mostly localized to the anterior labial gingiva, affecting females more than males. These types of melanin pigmentations are not considered as a medical problem or disease, and their impact is mainly on the aesthetic and social functioning particularly with females where aesthetic demands are higher [2].

Hence, pigmentation generally can be classified into the following categories:

(i) Inherited:
  1. Racial
(ii) Acquired:
  1. Postinflammatory pigmentation
  2. Melanocytic nevus
  3. Blue nevus, hemangioma, and hematoma
  4. Smoker's melanosis
  5. Oral melanoacanthoma
  6. Peutz–Jeghers syndrome
  7. Addison disease
  8. Drug, hormones, and chemical-induced pigmentations
  9. Hemangioma, hematoma
  10. Metal tattoo

The next level of differentiation is endogenous (melanin, hemosiderin, lipofuscin, and carotene) or exogenous (metals) origin of pigments. Especially the last-mentioned one is very frequent and very interesting for monitoring treatment.

Etiology and pathogenesis of oral pigmentations is very diverse, and lasers enable individual access according to the tissue pathology.

Histologically, melanogenesis is the oxidation of the tyrosine amino acid followed by polymerization to produce the melanin pigmentation in the melanocytes.

Melanin and melanocytes in gingiva are present in the epithelium, and the deepest possible location could reach the basal line [2].

Melanocyte cell numbers are similar in all humans, but the activity differs from one person to another and when activated more, melanin produced in the tissues results in darker pigmentation.

Pigmented malignant lesions show an increase of melanocyte number, whereas in benign pigment lesions, the activity of melanocytes increases and melanin production also increases [2, 3].

Many factors can induce the melanin production; environmental effects can lead to activation of melanocytes, including smoking [5–8], some medications, chemicals, and even unprotected gingiva during light whitening procedures, as whitening light can activate melanin production [2, 9]. Some light sources can induce the melanin production like excimer light and uvb lamps [10].

Many techniques have been used to remove the melanin pigmentation with different outcomes and factors such as patient comfort, postop care, and recurrence.

Scalpel, diamond bur, ceramic bur, chemicals, and deferent type of lasers have been used with acceptable esthetical results. However, patient comfort, complications, minimal invasiveness, and postop care were the goals in finding a better technique.

Lasers with different wavelengths (Er Lasers, diodes lasers, CO_2_ lasers, and the new q-switched lasers) have been used easily with well-tolerated and good esthetical outcomes as minimal-invasive techniques, which have been recorded in many published papers [11–26].

As melanin is produced, it resides in the basal cell layer of the gingival epithelium [2], so in this study the laser will be used in de-epithelization (peeling of the epithelial layer).

The aim of this study is to compare the results and the outcomes of 3 laser wavelengths (445, 940, and 2780 nm) in melanin depigmentation, with 2 years follow up to check for the relapse of the pigmentations.

Outcome measures of comparisons include wound healing, postop pain, duration of procedure, bleeding, and gingival color improvement. Patient aesthetic perception is measured in terms of satisfaction with treatment outcome and improvement in gingival color.

## 2. Materials and Methods

**Figure 1:**
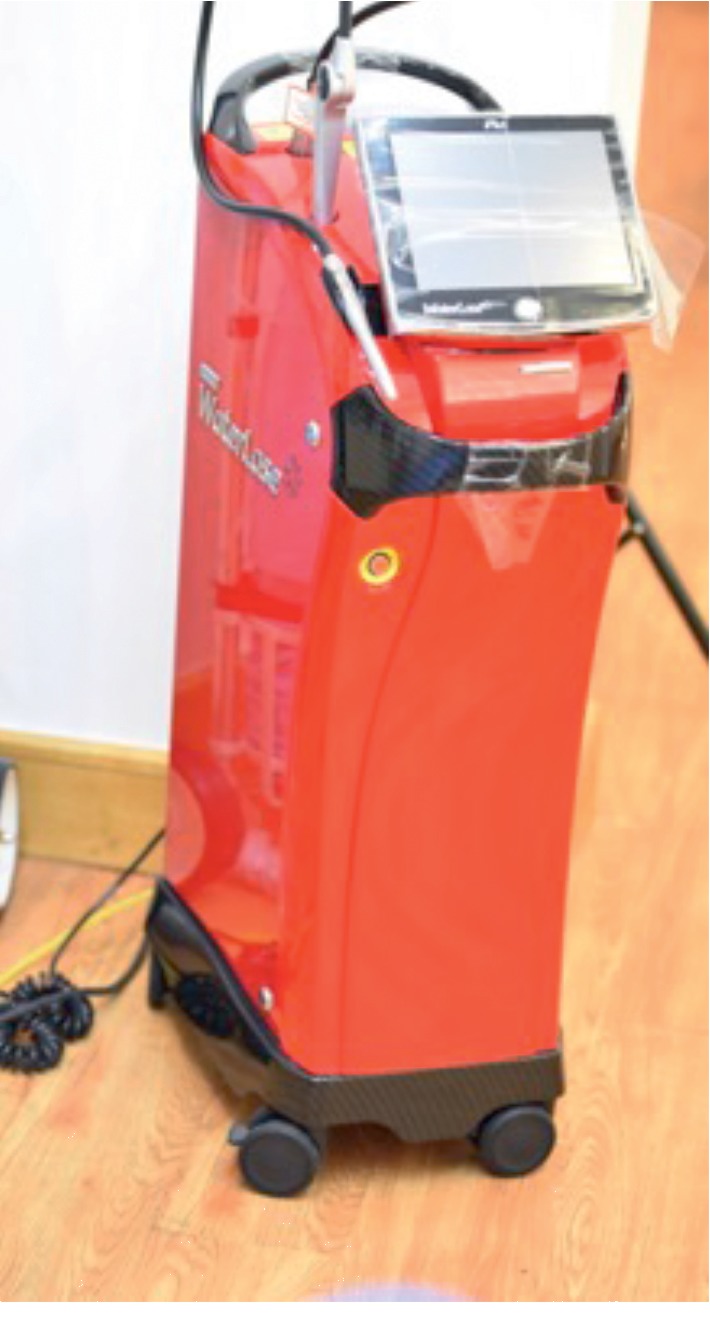
iPlus, Er, Cr: YSGG 2780 nm, from Biolase.

**Figure 2:**
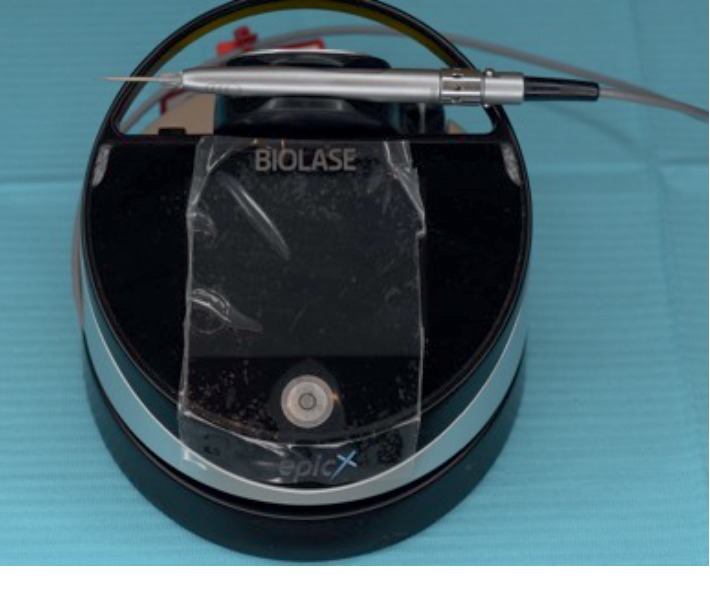
Epic, biolase 940 nm.

**Figure 3:**
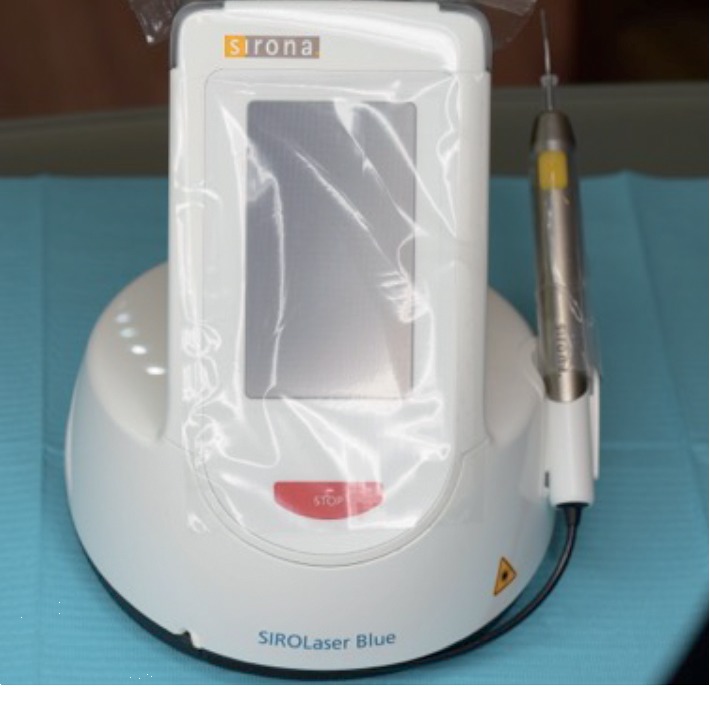
Sirolaser blue, Dentsply Sirona, 445 nm.

Three laser machines with different wavelengths have been used in this study:

1. Er, Cr: YSGG laser with 2780 nm (iPlus, Biolase, USA) (Figure 1)
2. Diode laser with 940 nm (Epic, Biolase, USA) (Figure 2)
3. Diode laser with 445 nm (Sirolase Blue, Dentsply Sirona, Germany) (Figure 3)

Patients have been selected and diagnosed, and the pigmentation underwent assessments using two indices:

Gingival pigmentation index GPI by Kumar et al. [27]:

- Score 0: absence of pigmentation
- Score 1: spots of brown to black color or pigments.
- Score 2: brown to black patches but not diffuse pigmentation
- Score 3: diffuse brown to black pigmentation, marginal, and attached

Melanin pigmentation index MPI by Hanioka et al. [28]:

- Score 0: no pigmentation
- Score 1: solitary unit(s) of pigmentation in papillary gingiva without extension between neighboring solitary units
- Score 2: formation of continuous ribbon extending from neighboring solitary units.

### 2.1. Outcome Measurements

1. Clinical parameters: bleeding, wound healing, pain, and duration of procedure were assessed at time and 10 days after procedure. Color improvements were assessed after 2 weeks of intervention.
2. Aesthetic parameters: photographs of smile taken before and after, and then they were assessed, by three calibrated cosmetic dentists; the procedure is blinded and randomized and repeated for validity and repeatability. Dentists classified ratings on a five-point smile improvement scale.
3. Perception parameters: satisfaction and quality of life parameters after 6 months of treatment.

### 2.2. A Simple Sampling Technique Was Utilized for Sample Selection

An equal number of cases were allocated to each type of laser used (Er, Cr: YSGG, and two wavelengths diode lasers).

A total of 30 cases were included (10 cases in each group):

  Group I: Er, CR:YSGG laser with (2.0 W, 22.2 J/cm^2^, 30 Hz, 30% air, 40% water) using MC3 chisel tip in contact mode (*n* = 10)
  Group II: diode laser 940 nm, with (800 mW, 636.9 J/cm^2^, continuous mode) using cylinder-shaped 400 *μ*m tip in contact mode (*n* = 10)
  Group III: diode laser 445 nm, with (300 mW, 424 J/cm^2^, continuous mode) using cylinder-shaped 320 *μ*m tip in contact mode (*n* = 10)

A multivariate descriptive analysis was used to demonstrate objective and subjective parameters in the study, and analysis of variance was used to compare the difference between the three methods of treatments (Statistical Package for the Social Sciences (SPSS) version 20.0; test names: chi-squared test and exact Fisher test for the descriptive evaluation of the data and Kruskal–Wallis test to compare between the groups using 95% confidence interval at *P* value <0.05).

### 2.3. Indices of Measurements

A: duration of the procedure using Er, Cr:YSGG and diode lasers was recorded in minutes to calculate the time precisely, and all the cases were a score 2 according to Melanin pigmentation index MPI by Hanioka et al.
Clinical parameters:
B: bleeding was scored (directly after surgery) on four-scale index
    0: no bleeding
    1: mild bleeding
    2: moderate bleeding
    3: severe bleeding
C: wound healing was scored (10 days after treatment) on 2-scale index
    0: complete healing
    1: partial healing (incomplete epithelial formation)
D: pain after the procedure was scored using VAS index of pain measurement:
    0: No pain ascending to 10—severe disabling pain
F: color improvement objective recording of gingival color variation using a five-point smile improvement scale
    0: no improvement
    1: little improvement
    2: average improvement
    3: great improvement
    4: complete improvement (natural gingival color including orange peel look)
G: satisfaction with treatment was documented depending on the patient's opinion, measured on a five-point scale after 6 months of treatment
    0: very dissatisfied
    1: dissatisfied
    2: neither satisfied nor dissatisfied
    3: satisfied
    4: very satisfied
H: relapse measured after 2 years “photo documentation”
    0: no relapse = no pigmentation at all
    1: mild relapse = mild pigmentation (separated islands)
    2: medium relapse = pigmentation is obvious and forming a small strip
    3: severe relapse = pigmentation is obvious and forming a wide strip

## 3. Results

### 3.1. Duration of the Procedure Measured in Minutes

**Figure 4:**
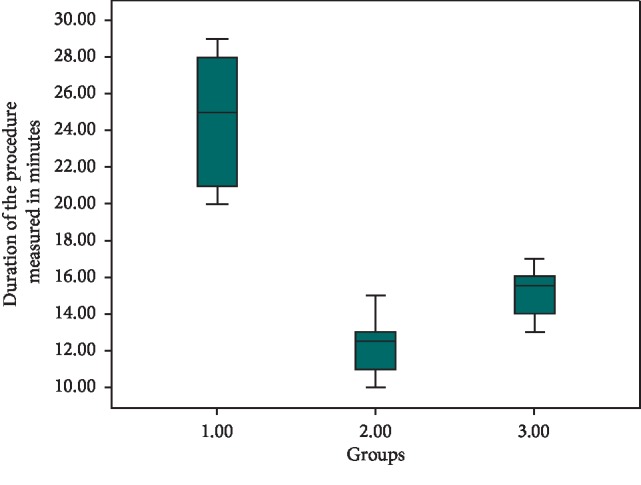
Duration of the procedure in minutes for the three treatment groups.

The duration needed to complete the procedures using the three laser units is shown in Table 1 and Figure 4. Statistical analysis showed a significant difference between the groups, in which group 1 was the most time-consuming procedure with an average mean of 24.6 minutes followed by group 3 with 15.1 minutes and the most time-effective procedure was group 2 with a mean value of 12.4 minutes.

### 3.2. Bleeding Results Directly after Surgery

**Figure 5:**
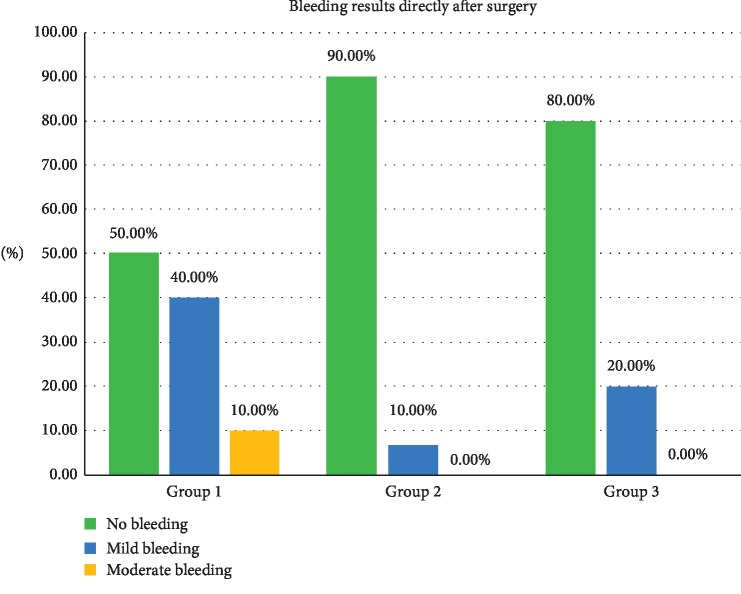
Bleeding frequency among the three procedures and its percentages.

Bleeding during the treatment for the three groups is presented in Table 2 and Figure 5. Cross tabulation showed that 90% of the patients treated with the diode 940 nm (group 2 laser) had no bleeding after the surgery comparing to only 10% experienced mild bleeding, followed by 80% treated with the diode 445 nm (group 3 laser), while only 20% reported with mild bleeding; on the other hand, 50% of the patients revealed no bleeding after the procedure with Er, Cr, YSGG 2780 nm (group 1 laser), while 40% had mild bleeding and 10% showed moderate bleeding after the surgery, but statistically there is no difference between the groups (*P* < 0.102).

### 3.3. Wound Healing 10 Days after Treatment

**Figure 6:**
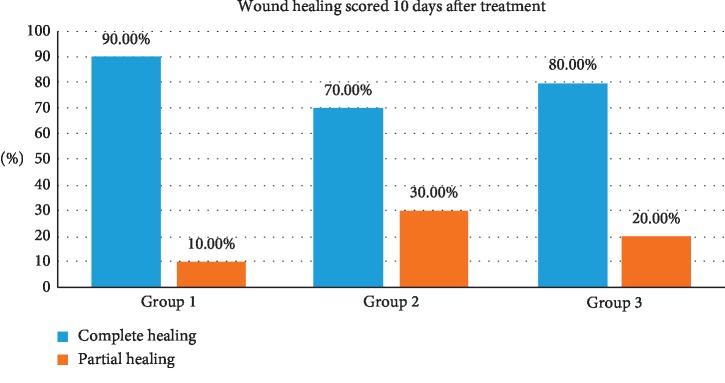
Number of healed cases among the three treatments scored 10 days after treatment.

The number of healed cases that has been scored 10 days after treatments is shown in Table 3 and Figure 6. Current study analysis revealed that more than two-third in all groups had complete healing 10 days following the treatment (90%, 70%, and 80%, respectively) with no significant differences between the groups (*P* < 0.547).

### 3.4. Pain Scored Using VAS Index of Pain Measurement

**Figure 7:**
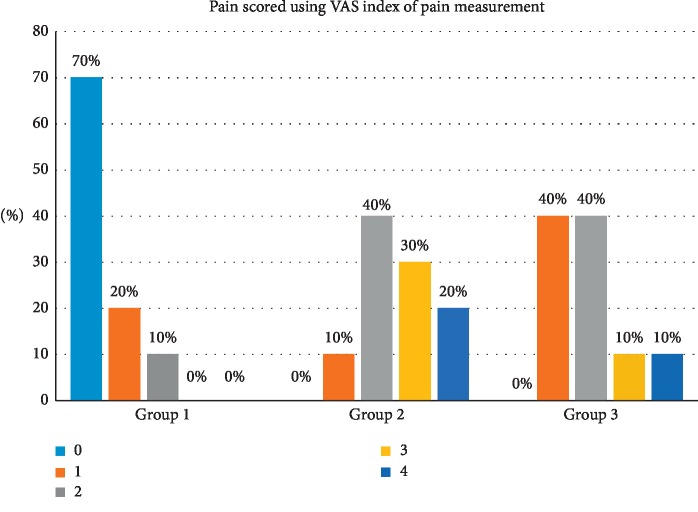
Pain after the procedure recorded by patients using the VAS scale.

The VAS index of pain is presented in Table 4 and Figure 7. After the procedures, relating to pain score, there was a significant difference between the groups, in which the majority of the patients (70%) had a painless experience after the procedure compared with 90% and 60% of (Group 2 and 3) that gave a pain score ranging from 2 to 4, respectively.

### 3.5. Color Improvement

**Figure 8:**
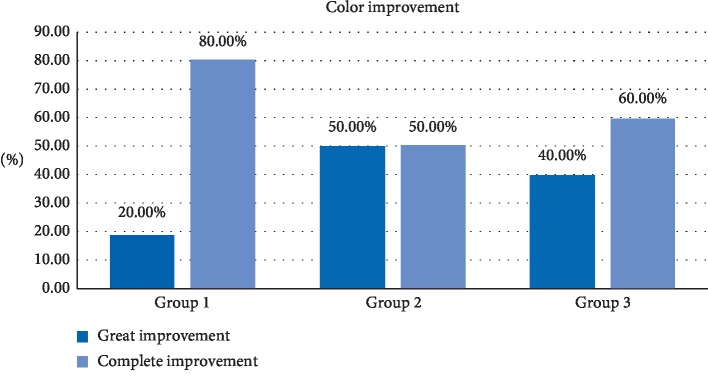
Color improvement for the three procedures.

Color improvement is presented in Table 5 and Figure 8. The current study revealed that color improvement within group 1 was the highest (80%) followed by group 3 (60%) and the least was group 2 (50%); yet, there was no statistically significant difference between groups (*P* < 0.379).

### 3.6. Satisfaction with Treatment Measured on a Five-Point Scale after 6 months of Treatment

**Figure 9:**
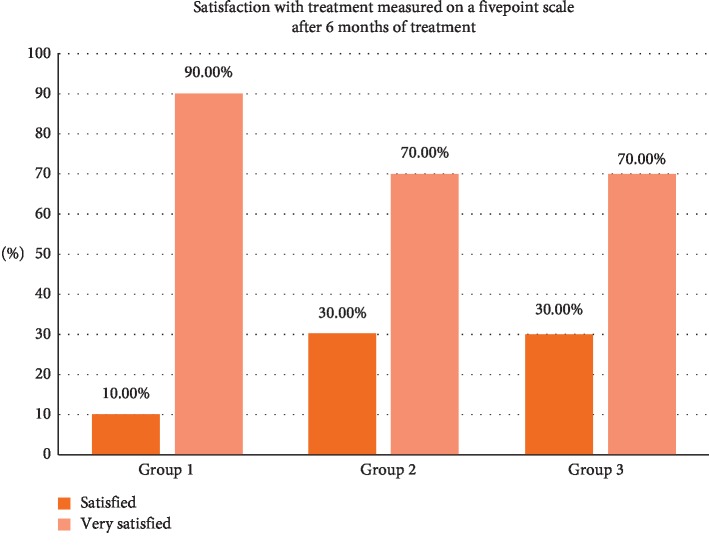
Satisfaction of the patient with results of treatment.

Patient satisfaction is presented in Table 6 and Figure 9. Most of the participants within the 1^st^ group (90%) were very satisfied after laser treatment, while 70% within the 2^nd^ and 3^rd^ groups were satisfied after the current procedure with no statistically significant difference (*P* < 0.487).

### 3.7. Relapse after 2 Years (Photo Documentation)

**Figure 10:**
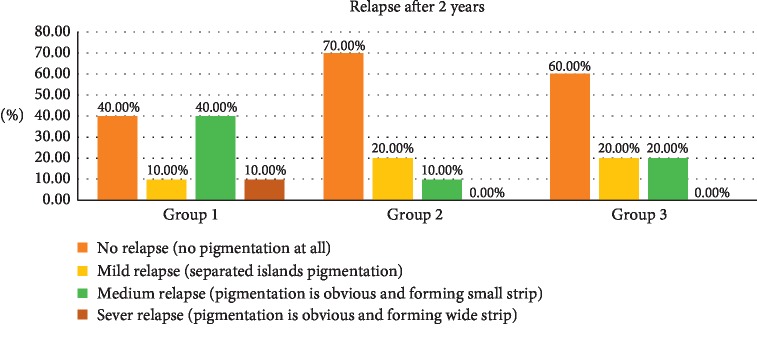
The relapse of the pigmentations documented after 2 years.

Photo documentation for relapse after two years is presented in Table 7 and Figure 10. Follow up after two years revealed that relapse mainly occurs within group 1, in which 40% of patients have had obvious pigmentation with small strip (medium relapse) and 10% equally experienced mild relapse and sever relapse. On the other hand, most of patients of 2^nd^ and 3^rd^ groups had no relapse within two years (70% and 60%, respectively). Statistically, still there are no significant differences (*P* < 0.203). Statistical analysis of correlation is given in Table 8.

## 4. Discussion

Previous studies were used to evaluate the effect of laser gingival depigmentation using different parameters, techniques, and even different lasers and tools. Positive opinions and conclusion have been discussed. Some papers suggest that the laser is an effective tool in gingival depigmentation specially when used by an experienced operator [8, 11–25, 29]. This study meant to check 3 different laser wavelengths and to clarify the different outcomes among them, especially as diode laser users have increased in the past few years.

According to the findings, the duration of the procedure was faster using the diode than the erbium group laser, and it is understandable that laser tissue interaction shows high absorption of the diodes wavelengths in the melanin pigmentation, and on the other hand, the erbium laser beam is much less absorbed by the melanin [30]. The high absorption of the diodes in pigmentations resulted in a faster peeling of the melanin, which reduced the time needed to complete the procedure. Significant differences between the groups (*P* < 0.000) in which the erbium laser group was the most time-consuming procedure with an average mean of 24.6 minutes followed by 445 nm group for 15.1 minutes, and the most time-effective procedure was 940 nm group with a mean of 12.4 minutes (Table 1, Figure 4).

The bleeding results in the procedure showed minimal to no bleeding using the three wavelengths; nevertheless, that minimal bleeding occurred with the erbium laser group as the penetration of this wavelength is less than in the two diodes. This is justified as the diodes have deeper penetration and thermal impact on the tissue which leads to more coagulation efficacy [31]. These results agree with many papers that used the lasers and notified that the high control in bleeding is achieved when using them [11–25, 32]. Statistically, there was no difference between the comparative groups (*P* < 0.102). (Table 2, Figure 5).

**Figure 11:**
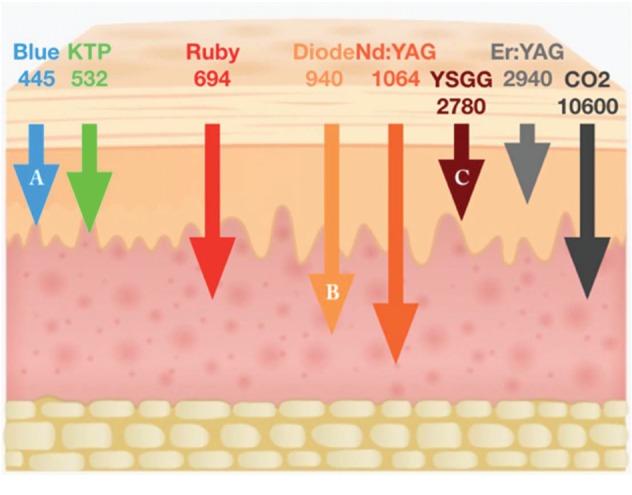
Penetration of different wavelengths in soft tissue, A is 445 nm, B is 940 nm, and C is Er, Cr, YSGG.

Healing was faster in the erbium laser group compared with the diodes, as diodes have more thermal impact on the tissue (Figure 11), which will be responsible for these results [31]. The thermal effect can delay the healing process, but it should be mentioned that, on the second recall, all the cases in all the groups were healed completely. Statistically, they were no significant differences between the groups (*P* < 0.547) (Table 3, Figure 6). These results concur with many previous papers where healing has been achievable using lasers with similar treatment procedures [11–25].

The pain results in this study showed a significant difference between the groups (*P* < 0.000) in which the majority of the patients (70%) had a painless experience after the procedure compared with 90% and 60% of patients treated by the 940 nm and 445 nm lasers. They gave a pain score ranging from 2 to 4, respectively, but with high patient acceptance and great tolerance for most of the cases, although the treatment by the erbium group laser was the least painful as it has the least thermal impact on the tissue [31], followed by the blue diode and then the infrared diode laser (Table 4, Figure 7). The erbium laser group pain results supports the work done by Hegde et al. that erbium laser was the least painful [23].

The color improvement varied from great to complete improvement in all the cases and that supports previous studies done and suggests that lasers are an effective tool in gingival depigmentation [11–25]; all the three lasers were effective in the enhancement of the color. The erbium laser gave a more aesthetic result as complete improvement despite that statistically there were no significant differences between the groups (Table 5, Figure 8).

The satisfaction results were between satisfied and very satisfied and that was according to the patients' total experience from day of the surgery to 6 months after surgery. Patients were happy with the results and expressed their satisfaction for the whole experience. No statistically significant difference (*P* < 0.487) between the three lasers has been noted (Table 6, Figure 9).

The melanin depigmentation is in the Gulf region considered as racial, and as it is impossible to control the activity of the melanocytes, this makes the relapse more likely to happen with time. Reducing the impact of other factors like smoking, some medications, and some habits may prolong the stability of the results. This study is planned to have data about relapse, after 2 years of follow up. Many publications have mentioned recurrence of the melanin pigmentation [23–25]. It is known that melanin production is activated by many factors as mentioned above, hence, to reduce the relapse possibility and prolong the time of the results, patients should avoid those factors especially smoking. [5–8].

The relapse was higher in the erbium laser group and lower in the diode groups. Diodes are well absorbed in the melanin pigmentation as mentioned, and the diodes are more penetrating in the tissue depth, which probably reduces the possible presence of nonpeeled pigmented cells or particles. The results of this study again agree with Hegde et al.'s results that the erbium laser has the higher rate of relapse. [23].

## 5. Conclusion

  Within the limitation of this study, all three wavelengths were fast, effective in peeling the pigmentations, and well tolerated by the patients. The aesthetic results were really positive, and the patients were highly satisfied.
  It is suggested to have a bigger number of samples in the future papers, and histological studies could be included to explore the different impact of each wavelength on the gingival melanin pigmentation specifically and on the gingival tissue generally.

## Figures and Tables

**Table 1:** Results of the time recorded of duration for the procedure in minutes.

	Minimum	Maximum	Mean ± SD
Group 1	20.00	29.00	24.60 ± 3.66
Group 2	10.00	15.00	12.40 ± 1.50
Group 3	13.00	17.00	15.10 ± 1.45
Total	10.00	29.00	17.37 ± 5.82

**Table 2:** Bleeding during the procedure for each group, note that both the diode groups (940 nm and 445 nm) exhibited less bleeding than the Er, Cr, YSGG 2780 (group 1).

			Bleeding results directly after surgery			Total
			No bleeding	Mild bleeding	Moderate bleeding	
Groups	1.00	Count	5	4	1	10
		% within groups	50.0%	40.0%	10.0%	100.0%
	2.00	Count	9	1	0	10
		% within groups	90.0%	10.0%	0.0%	100.0%
	3.00	Count	8	2	0	10
		% within groups	80.0%	20.0%	0.0%	100.0%

Total		Count	22	7	1	30
		% within groups	73.3%	23.3%	3.3%	100.0%

**Table 3:** Healing documented in the 1^st^ recall (after 10 days), note that all the cases have been completely healed in the 2^nd^ patient recall.

			Wound healing is scored (10 days after treatment)		Total
			Complete healing	Partial healing	
Groups	1.00	Count	9	1	10
		% within groups	90.0%	10.0%	100.0%
	2.00	Count	7	3	10
		% within groups	70.0%	30.0%	100.0%
	3.00	Count	8	2	10
		% within groups	80.0%	20.0%	100.0%

Total		Count	24	6	30
		% within groups	80.0%	20.0%	100.0%

**Table 4:** Pain after the procedure recorded by patients using the VAS scale.

			Pain is scored using VAS index of pain measurement					Total
			0.00	1.00	2.00	3.00	4.00	
Groups	1.00	Count	7	2	1	0	0	10
		% within groups	70.0%	20.0%	10.0%	0.0%	0.0%	100.0%
	2.00	Count	0	1	4	3	2	10
		% within groups	0.0%	10.0%	40.0%	30.0%	20.0%	100.0%
	3.00	Count	0	4	4	1	1	10
		% within groups	0.0%	40.0%	40.0%	10.0%	10.0%	100.0%

Total		Count	7	7	9	4	3	30
		% within groups	23.3%	23.3%	30.0%	13.3%	10.0%	100.0%

**Table 5:** Color improvement for the three procedures and its percentage.

			Color improvement		Total
			Great improvement	Complete improvement	
groups	1.00	Count	2	8	10
		% within groups	20.0	80.0	100.0
	2.00	Count	5	5	10
		% within groups	50.0	50.0	100.0
	3.00	Count	4	6	10
		% within groups	40.0	60.0	100.0

Total		Count	11	19	30
		% within groups	36.7	63.3	100.0

*Note.* The color was assessed by three calibrated cosmetic dentists. The procedure was blinded and randomized and repeated for validity and repeatability. Dentists classified ratings on a five-point smile improvement scale.

**Table 6:** Satisfaction of the patient with results of treatment.

			Satisfaction with treatment is measured on a five-point scale after 6 months of treatment		Total
			Satisfied	Very satisfied	
Groups	1.00	Count	1	9	10
		% within groups	10.0%	90.0%	100.0%
	2.00	Count	3	7	10
		% within groups	30.0%	70.0%	100.0%
	3.00	Count	3	7	10
		% within groups	30.0%	70.0%	100.0%

Total		Count	7	23	30
		% within groups	23.3%	76.7%	100.0%

**Table 7:** The relapse of the pigmentations documented after 2 years.

			Relapse after 2 years (photo documentation)				Total
			No relapse	Mild relapse	Medium relapse	Sever relapse	
groups	1.00	Count	4	1	4	1	10
		% within groups	40.0%	10.0%	40.0%	10.0%	100.0%
	2.00	Count	7	2	1	0	10
		% within groups	70.0%	20.0%	10.0%	0.0%	100.0%
	3.00	Count	6	2	2	0	10
		% within groups	60.0%	20.0%	20.0%	0.0%	100.0%

Total		Count	17	5	7	1	30
		% within groups	56.7%	16.7%	23.3%	3.3%	100.0%

**Table 8:** Statistical analysis of correlation (Kruskal–Wallis and chi-squared tests were also used to determine the correlation between the independent groups).

Test statistics							
	Bleeding directly after surgery	Color improvement	Pain is scored using VAS index	Satisfaction with treatment after 6 months	Wound healing after 10 days	Relapse after two years	Duration of the procedure measured in minutes
Chi-squared	4.573	1.943	16.651	1.441	1.208	3.191	23.679
Df	2	2	2	2	2	2	2
Asymp Sig.	0.102	0.379	0.000	0.487	0.547	0.203	0.000

## Data Availability

The data used to support the findings of this study are included within the article, and other data are available from the corresponding author upon request.
